# Supplementary material for: The Effect of the BCO2 Genotype on the Expression of Genes Related to Carotenoid, Retinol, and α-Tocopherol Metabolism in Rabbits Fed a Diet with Aztec Marigold Flower Extract
Source: Int J Mol Sci. 2022 Sep 11;23(18):10552. doi: 10.3390/ijms231810552 (PMC9506012; doi:10.3390/ijms231810552)
Supplement: Supplementary file 1 [file ijms-23-10552-s001.zip › Supplement2.pdf]

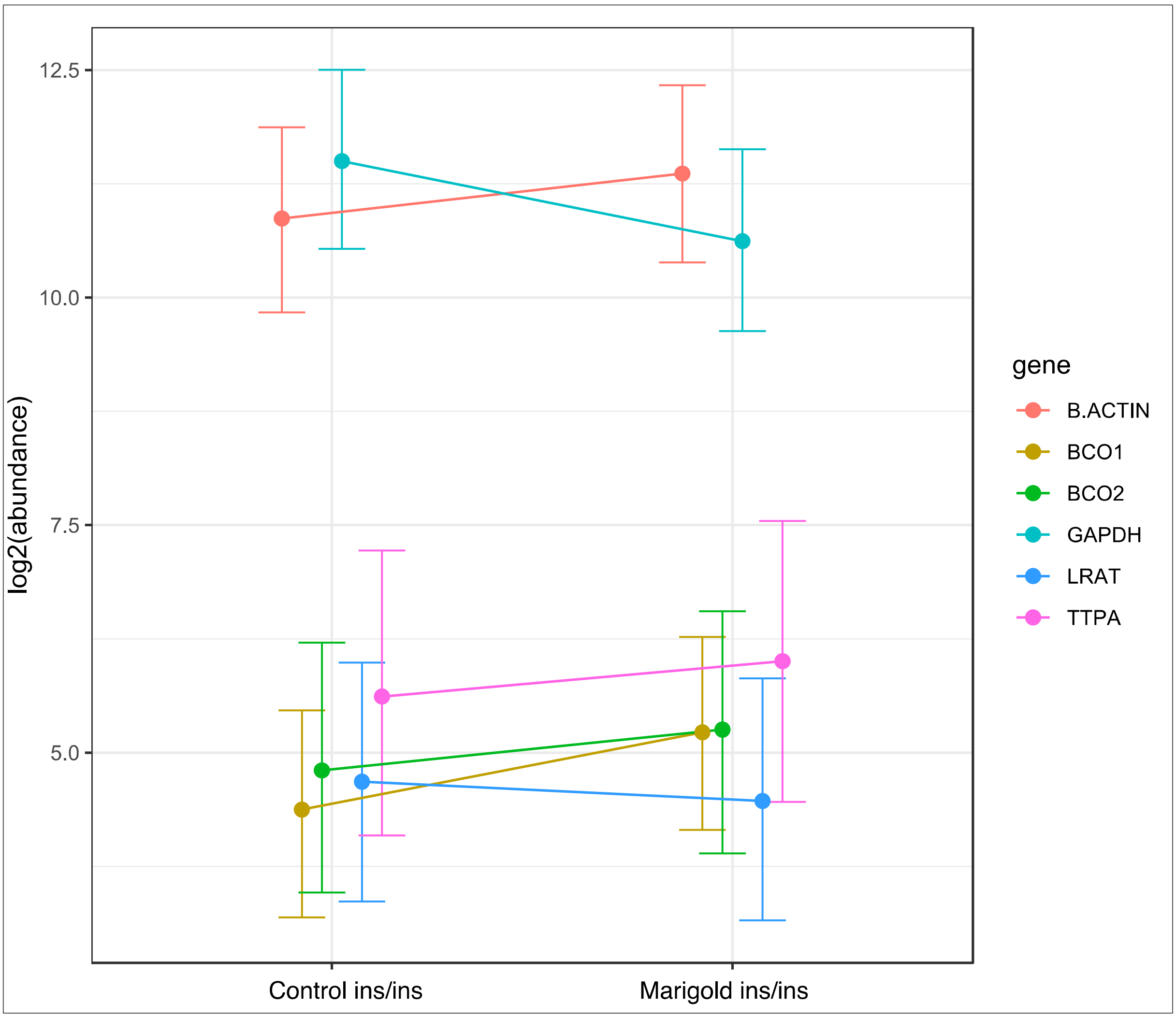

Figure S1. Relative BCO1, BCO2, LRAT and TTPA mRNA levels in liver of rabbits fed different diets (control diet vs. diet with the addition of Aztec marigold flower extract) having ins/ins genotype at codon 248 of the BCO2 gene. GAPDH and ß-actin were used as reference genes. Data represent the posterior means (expressed as arbitrary units) ± 95% credible intervals. No statistically significant differences were noted.

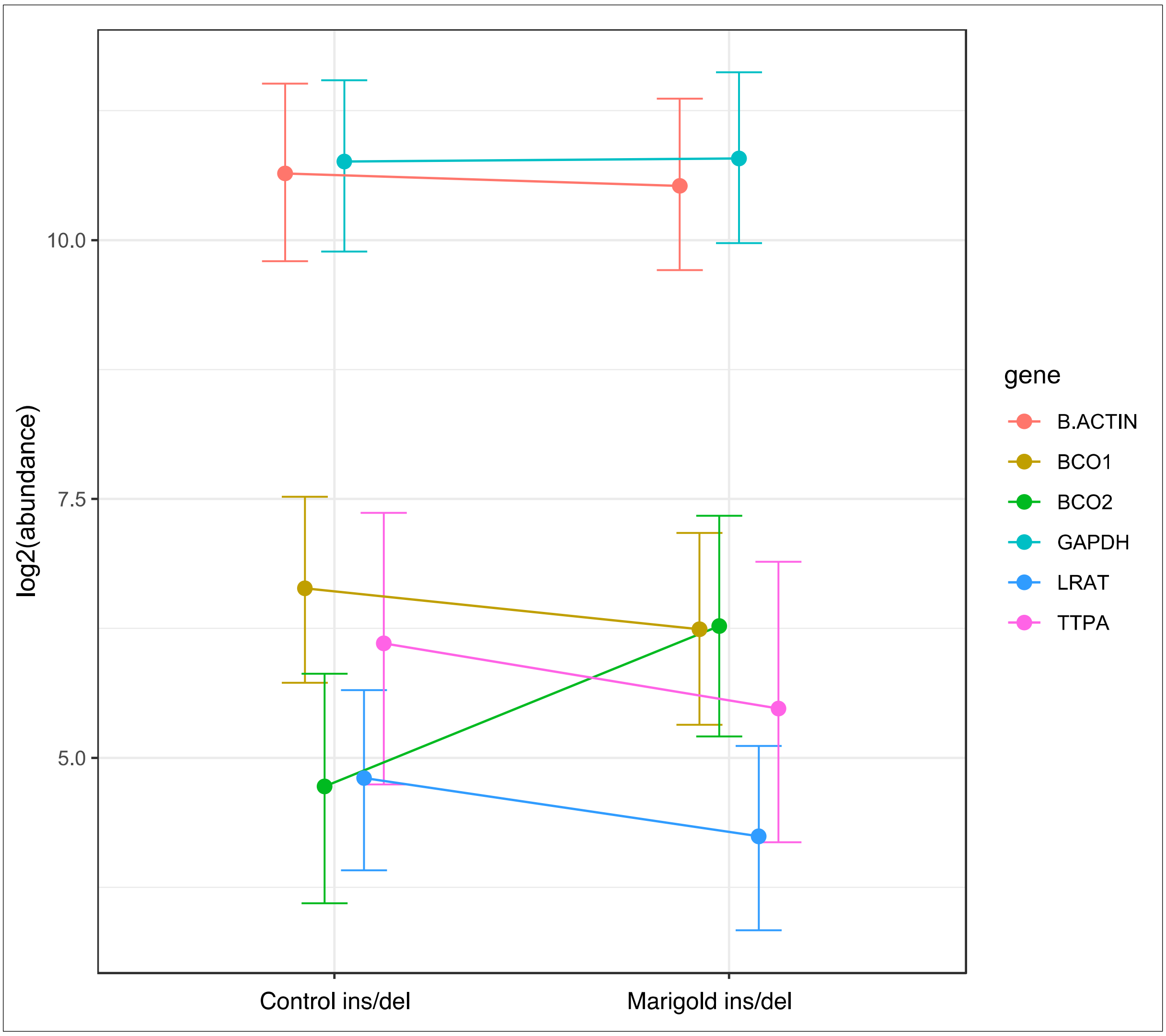

Figure S2. Relative BCO1, BCO2, LRAT and TTPA mRNA levels in liver of rabbits fed different diets (control diet vs. diet with the addition of Aztec marigold flower extract) having ins/del genotype at codon 248 of the BCO2 gene. GAPDH and ß-actin were used as reference genes. Data represent the posterior means (expressed as arbitrary units) ± 95% credible intervals. No statistically significant differences were noted.
